# *Coprinopsis cinerea* dioxygenase is an oxygenase forming 10(*S*)-hydroperoxide of linoleic acid, essential for mushroom alcohol, 1-octen-3-ol, synthesis

**DOI:** 10.1016/j.jbc.2022.102507

**Published:** 2022-09-17

**Authors:** Takuya Teshima, Risa Funai, Takehito Nakazawa, Junya Ito, Toshihiko Utsumi, Pattana Kakumyan, Hiromi Mukai, Toyoshi Yoshiga, Ryutaro Murakami, Kiyotaka Nakagawa, Yoichi Honda, Kenji Matsui

**Affiliations:** 1 Graduate School of Sciences and Technology for Innovation, Yamaguchi University, Yamaguchi, Japan; 2 Graduate School of Agriculture, Kyoto University, Kyoto, Japan; 3 Food and Biodynamic Chemistry Laboratory, Graduate School of Agricultural Science, Tohoku University, Sendai, Miyagi, Japan; 4 School of Science, Mae Fah Luang University, Chiang Rai, Thailand; 5 Department of Forest Entomology, Forestry and Forest Products Research Institute, Tsukuba, Japan; 6 Department of Applied Biological Sciences, Faculty of Agriculture, Saga University, Saga, Japan

**Keywords:** mushroom, fatty acid metabolism, dioxygenase, cytochrome P450, oxylipins, 1-octen-3-ol, fungi-fungivore interaction, cDNA, complementary DNA, MTBE, methyl *tert*-butyl ether, PDA, potato dextrose agar, qPCR, quantitative PCR, SPME, solid-phase microextraction, YMG, yeast extract-malt extract-glucose

## Abstract

1-Octen-3-ol is a volatile oxylipin found ubiquitously in Basidiomycota and Ascomycota. The biosynthetic pathway forming 1-octen-3-ol from linoleic acid *via* the linoleic acid 10(*S*)-hydroperoxide was characterized 40 years ago in mushrooms, yet the enzymes involved are not identified. The *dioxygenase 1* and *2* genes (*Ccdox1* and *Ccdox2*) in the mushroom *Coprinopsis cinerea* contain an N-terminal cyclooxygenase-like heme peroxidase domain and a C-terminal cytochrome P450-related domain. Herein, we show that recombinant CcDOX1 is responsible for dioxygenation of linoleic acid to form the 10(*S*)-hydroperoxide, the first step in 1-octen-3-ol synthesis, whereas CcDOX2 conceivably forms linoleic acid 8-hydroperoxide. We demonstrate that KO of the *Ccdox1* gene suppressed 1-octen-3-ol synthesis, although added linoleic acid 10(*S*)-hydroperoxide was still efficiently converted. The P450-related domain of CcDOX1 lacks the characteristic Cys heme ligand and the evidence indicates that a second uncharacterized enzyme converts the 10(*S*)-hydroperoxide to 1-octen-3-ol. Additionally, we determined the gene KO strain (Δ*Ccdox1*) was less attractive to fruit fly larvae, while the feeding behavior of fungus gnats on Δ*Ccdox1* mycelia showed little difference from that on the mycelia of the WT strain. The proliferation of fungivorous nematodes on Δ*Ccdox1* mycelia was similar to or slightly worse than that on WT mycelia. Thus, 1-octen-3-ol seems to be an attractive compound involved in emitter–receiver ecological communication in mushrooms.

1-Octen-3-ol is a volatile compound with an earthy and mushroom-like organoleptic property that commonly occurs in nature, such as in mushrooms, molds, and moist air in a laurel forest. It is also found in human breath and sweat. It attracts biting insects, such as mosquitoes (1). An olfactory receptor specific to 1-octen-3-ol has been isolated from malaria-causing mosquitoes (2). Despite its familiarity, the details of the biosynthesis of 1-octen-3-ol, as well as its ecological and physiological significance, are not fully understood.

The biosynthetic pathway for 1-octen-3-ol formation in common mushrooms (*Agaricus bisporus*) has been elucidated by Wurzenberger and Grosch (3, 4, 5). Linoleic acid is the substrate, and its stereospecific oxygenation yields the 10(*S*)-hydroperoxide of linoleic acid (10(*S*)HPODE) and subsequent cleavage yields (*R*)-(-)-1-octen-3-ol and 10-oxo-(*E*)-9-decenoic acid (Fig. 1). The involvement of the 10(*S*)-isomer as an intermediate to form 1-octen-3-ol from linoleic acid was also confirmed in *Lentinula edodes* (Shiitake mushroom) and *Tricholoma matsutake* (Matsutake mushroom) (6, 7). However, despite the efforts of many researchers, the enzymes involved in this biosynthetic pathway have not been identified for 40 years.

**Figure 1:**
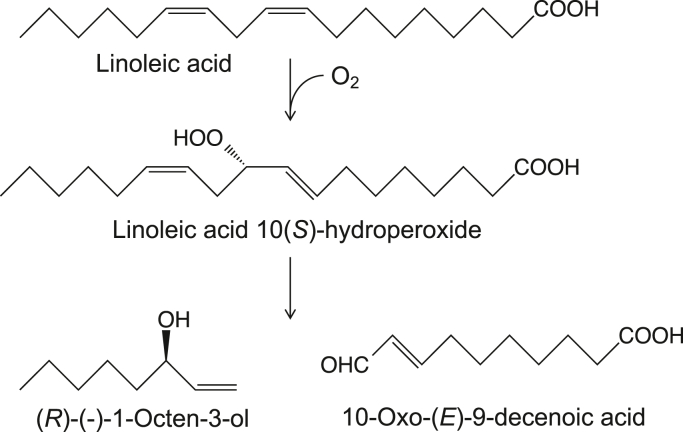
**Pathway for the biosynthesis of 1-octen-3-ol from linoleic acid in Basidiomycota**.

Filamentous Ascomycota produce an array of oxylipin metabolites called precocious sexual inducer (psi) factors, which function as hormone-like signals. Psi-producing oxygenases (Ppos) are responsible for the biosynthesis of psi factors (8, 9, 10, 11). Each Ppo is a fusion protein consisting of a cyclooxygenase (COX)-like domain at its N terminus and cytochrome P450-like domain at the C terminus. The COX domain catalyzes a dioxygenase (DOX) reaction with unsaturated fatty acids, such as linoleic acid and oleic acid, and the P450-like domain catalyzes a rearrangement reaction with the fatty acid hydroperoxide formed by the COX domain (10, 11). The filamentous Ascomycota have several subtypes of Ppos, such as PpoA, B, C, or D, which show diversity in the position of oxygenation on fatty acid substrates, catalyzed by the COX domain and the mode of the rearrangement of hydroperoxides thus formed, which is catalyzed by the P450 domain (10, 11). For example, PpoA, also called linoleate diol synthase, converts linoleic acid to its 8-hydroperoxide, which subsequently isomerizes to form 5,8-dihydroxy or 7,8-dihydroxy linoleic acid (12). PpoC from Aspergilli also consists of an N-terminal COX domain and C-terminal P450-like domain. The COX domain accounts for the formation of the 10-hydroperoxide of linoleic acid but the P450-like domain does not rearrange hydroperoxide; thus, PpoC almost exclusively forms the 10-hydroperoxide of linoleic acid (13). Because of the same position of the hydroperoxide group in linoleic acid, PpoC-like enzymes are expected to be involved in the production of 1-octen-3-ol in filamentous Ascomycota. Disruption of *ppoC*-like genes in *Podospora anserina* and *Aspergillus luchuensis* diminishes their ability to produce 1-octen-3-ol (14, 15). However, it has not been confirmed whether the *ppoC*-like genes of *P. anserina* and *A. luchuensis* are directly involved in the formation of 1-octen-3-ol because the catalytic properties of these gene products have not been studied. Furthermore, the configuration of the PpoC reaction products from filamentous ascomycetes analyzed to date was 10(*R*)-hydroperoxide (7), which was not cleaved to form 1-octen-3-ol by the crude extract prepared from common mushrooms (5).

To date, four genes with substantial similarities with ascomycete *ppo*s have been reported in Basidiomycota, such as *Ustilago maydis* and *Rhizoctonia solani* (16, 17); however, the properties and functions of the enzymes encoded by these basidiomycete genes have never been studied. In this study, we identified two genes, *Ccdox1* and *Ccdox2*, in the genome of the model basidiomycete *Coprinopsis cinerea*, which shares homology with *ppo*s from filamentous ascomycetes. *C. cinerea*, commonly known as the gray shag, is a model multicellular basidiomycete that completes its entire life cycle through a sexual cycle within 2 weeks in the laboratory (18). A rich genetic resource, with genome sequences, morphological and developmental mutants, and DNA markers, is available for the mushroom. *C. cinerea* is relatively easy to genetically transform, and a procedure for targeted gene disruption has been established (19). We characterized the enzymatic properties of recombinant CcDOX1 and CcDOX2 expressed in insect cells. *Ccdox1* gene was disrupted through homologous recombination to confirm its involvement in 1-octen-3-ol formation.

The ecophysiological role of 1-octen-3-ol may confer the benefit of sporophyte dispersal by attracting mosquitoes and flies. 1-Octen-3-ol has also been reported to have behavioral suppression and repellent effects on several arthropods and nematodes (20), indicating its potential involvement in the defense against fungivores. However, there is no clear evidence confirming the contribution of 1-octen-3-ol to Basidiomycota. In this study, a *C. cinerea* strain deficient in the ability to form 1-octen-3-ol was used to examine the ecological and/or physiological significance of 1-octen-3-ol.

## Results

### 1-Octen-3-ol formation in *C. cinerea*

**Figure 2:**
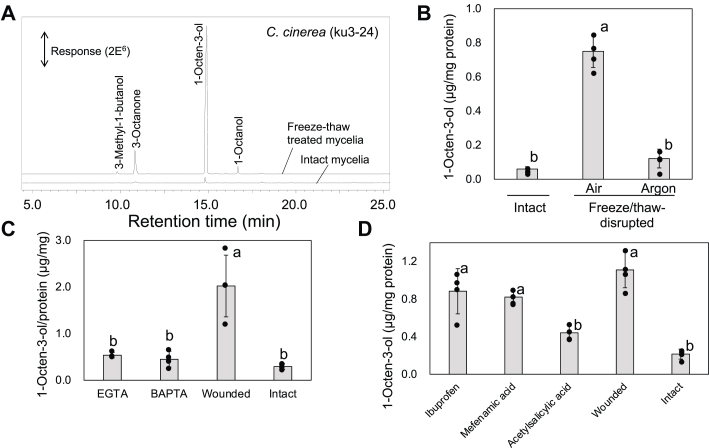
**Formation of 1-octen-3-ol by the mycelia of *Coprinopsis cinerea* (strain ku3-24).***A*, representative chromatograms of the volatiles formed from intact and freeze-thaw–treated mycelia. The volatiles in the headspace of a vial containing intact and freeze-thaw treated mycelia were collected with an SPME fiber. Effect of anaerobic condition (*B*), Ca^2+^-chelating reagents (EGTA and BAPTA, at 1 mM) (*C*), and cyclooxygenase inhibitors (at 5 μM) (*D*) on 1-octen-3-ol formation accelerated by disrupting the mycelia. For these analyses, 1-octen-3-ol was extracted with MTBE for quantification with GC-MS analyses. The amount of 1-octen-3-ol is presented as mean ± SD (error bar, *n* = 3). The different letters indicate significant differences, as identified using one way ANOVA and least squares method; *p* < 0.05. MTBE, methyl *tert*-butyl ether; SPME, solid-phase microextraction.

1-Octen-3-ol was barely detected in the intact mycelia of *C. cinerea* (strain ku3-24) grown on yeast extract-malt extract-glucose (YMG) agar plates, but the formation of considerable amounts of 1-octen-3-ol along with 3-octanone, 1-octanol, and 3-methylbutanal was observed within 30 min of damaging the mycelia with freeze-thaw treatment (Fig. 2*A*). The rapid formation of 1-octen-3-ol was effectively suppressed in the absence of molecular oxygen (Fig. 2*B*), indicating *de novo* biosynthesis of 1-octen-3-ol from linoleic acid. Rapid formation of 1-octen-3-ol was also observed when the mycelia were disrupted with a bead crusher, and the formation was suppressed by the addition of Ca^2+^ chelating reagents, such as BAPTA or EGTA (Fig. 2*C*). Acetylsalicylic acid, a typical cyclooxygenase inhibitor, also suppressed 1-octen-3-ol formation; however, only minimal suppression was observed with the cyclooxygenase inhibitors ibuprofen and mefenamic acid (Fig. 2*D*).

### *C. cinerea* dioxygenases

**Figure 3:**
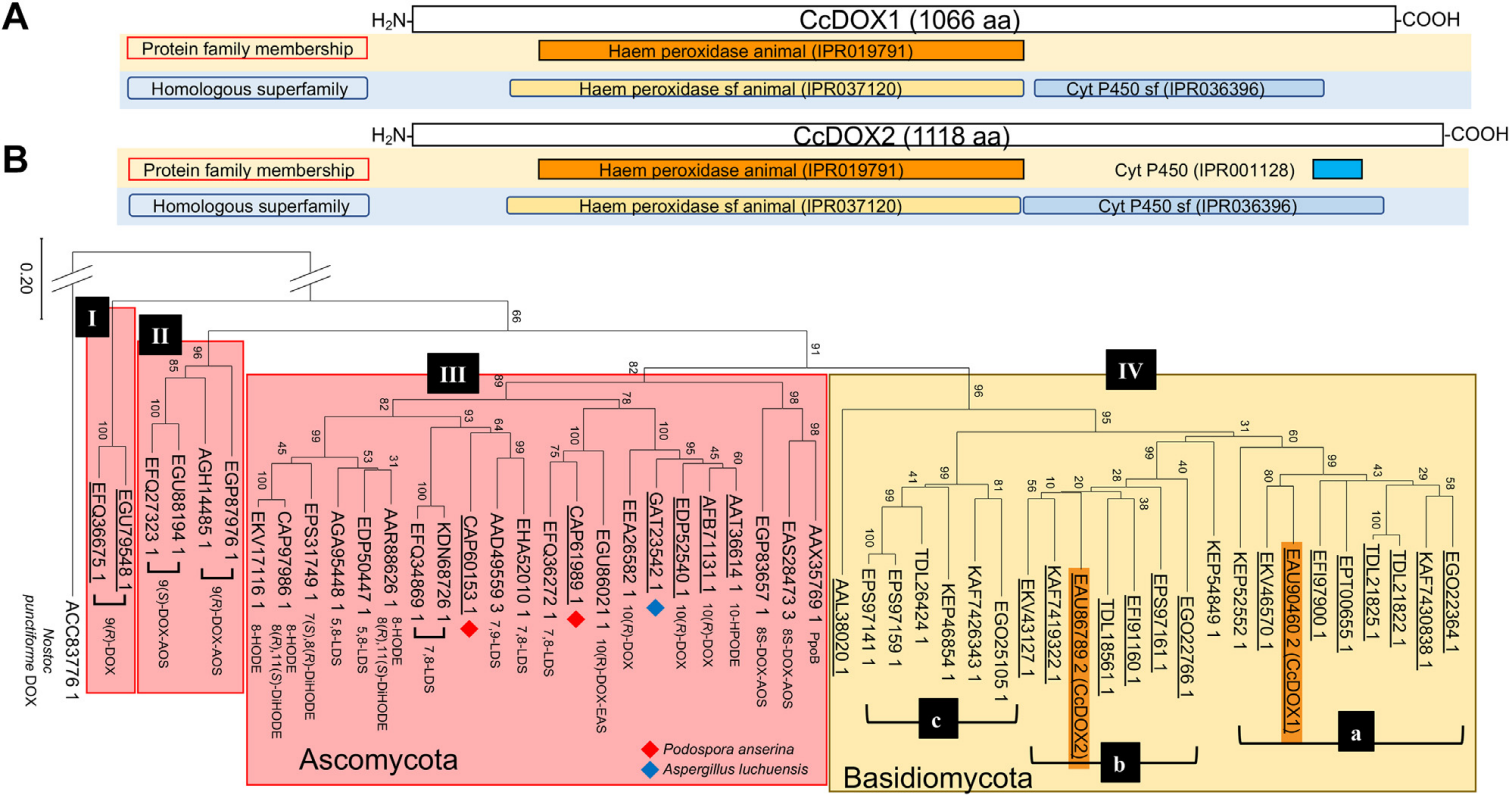
**Characterization of CcDOX1 and CcDOX2 sequences.***A*, schematic representation of their domain structures identified with InterProScan. The two categories, protein family category and homologous superfamily, are shown. *B*, phylogenetic analysis of CcDOX1 and CcDOX2 with the DOX-related proteins from different species belonging to Ascomycota and Basidiomycota. Phylogenetic analysis was performed with MEGA X using the maximum likelihood method. The details of the sequences used here are shown in Table S1. CcDOX1 and CcDOX2 are highlighted with an *orange background*. The proteins encoded by *ppoC*-like genes that were proposed to be involved in 1-octen-3-ol formation in *Podospora anserina* (*red diamonds*) and *Aspergillus luchuensis* (*blue diamond*) are also highlighted. The proteins lacking either or both the His and the Cys in the heme signature motif (FxxGx(H/R)xCxG motif) are underlined. The cyanobacterial (*Nostoc punctiforme*) DOX is used as the outgroup. Each clade is named as I, II, III, and IV. The clade IV is further divided into three sub-clades, a, b, and c.

Because Ppo and lipoxygenase (LOX) have been reported in fungi as enzymes catalyzing the dioxygenation of fatty acids to yield the hydroperoxide derivative (10), we assumed that an enzyme similar to Ppo or LOX participated in the first fatty acid oxygenation step in the biosynthetic pathway to form 1-octen-3-ol. When manganese LOX in the filamentous ascomycete *Gaeumannomyces graminis* var*. avenae* (oat-take-all root rot fungus) (GenBank, AAK81882.2) (21) was used as a query for BLASTP search with the *C. cinerea* genome database (*Coprinopsis cinerea* AmutBmut pab1-1 v1.0 in MycoCosm) (22), no protein with a substantial similarity was detected. When the same BLASTP search was performed with *Aspergillus nidulans* PpoC protein (AAT36614) as the query, two proteins with protein IDs 423716 and 398037 (GenBank: EAU90460.2 and EAU86789.2, respectively) were found, with E-values smaller than 1.0e^−100^. They were tentatively named CcDOX1 and CcDOX2. CcDOX1 and CcDOX2 are 1066 and 1118 amino acids long, respectively, and share 38% identity and 54% homology. TargetP-2.0 analyses (http://www.cbs.dtu.dk/services/TargetP/) showed that both proteins contained neither a signal peptide nor a mitochondrial transit peptide. InterProScan (https://www.ebi.ac.uk/interpro/search/sequence/) with CcDOX1 indicated that it consists of an N-terminal domain belonging to the animal heme peroxidase family (IPR037120) and a C-terminal domain that was classified as a sequence homologous to the cytochrome P450 superfamily (IPR036396), although the sequence was not classified as a member of any protein family specified in InterPro (Fig. 3*A*). This was mostly the case with CcDOX2, but a short segment of its C-terminal domain was classified as a member of the cytochrome P450 family (IPR001128) (Fig. 3*A*). The catalytic Tyr (Tyr-395 and Tyr-407 in CcDOX1 and CcDOX2, respectively) and distal and proximal His (His-217/His-398 and His-227/His-410 in CcDOX1 and CcDOX2, respectively) essential for binding heme are highly conserved as found with animal COXs (23) (Fig. S1). They are conserved at the apparently suitable positions in the protein sequences in the N-terminal domains of CcDOX1/2. The Arg residue critical for binding fatty acid substrates (Arg-106 in mouse COX2) was not found, and Ser, which is the target of nonsteroidal anti-inflammatory drugs (Ser-516 in mouse COX2) (23), was replaced with Thr in CcDOX1/2 (Fig. S1).

The signature of the C-terminal domain of P450 is not distinct. The ExxR motif (24), which is one of the most conserved residues in the K-helix of Cyt P450 enzymes, is conserved in CcDOX1/2; however, the heme signature motif (FxxGx(H/R)xCxG motif) (24) is quite different in CcDOX1/2. For example, the His residue, which has been shown to be essential for hydroperoxide isomerase activity in *A. nidulans* PpoA (His1004) (24), and the Cys residue, crucial for P450 activity as the fifth heme iron ligand through the heme-thiolate bond (25), are replaced by Phe/Thr and Tyr/Leu in CcDOX1/2, respectively (Fig. S2). Substitution of these amino acid residues, which are essential for P450 catalysis, is also evident in *A. nidulans* PpoC, which has no ability to rearrange the fatty acid hydroperoxide depending on P450 catalysis (13). Hereafter, the C-terminal domain of CcDOX1/2 is referred to as a P450-related domain.

The CcDOX1 sequence was used as a query for BLASTP analyses in MycoCosm (22) against the protein databases of the genome sequences of representative species belonging to diverse classes in Basidiomycota, namely, *A. bisporus* (Agaricales), *Pleurotus ostreatus* (Agaricales), *Schizophyllum commune* (Agaricales), *R. solani* (Cantharellales) (17), *Serpula lacrymans* (Boletales), *Fomitopsis pinicola* (Polyporales), *Rickenella mellea* (Rickenella), and *U. maydis* (Ustilaginomycotina) (16). Proteins with a significant similarity (<1.0e^−5^) were chosen and a phylogenetic tree was constructed along with the protein sequences of representative DOX-P450 fusion proteins (Ppos) from Ascomycota (26) (Fig. 3*B*). The proteins were divided into four major clades (clade I to IV), three of which (clade I, II, and III) consisted of proteins in Ascomycota, and the other one (clade IV) consisted of those in Basidiomycota. The proteins found in Basidiomycota were further divided into three major clades (subclades a, b, and c), where CcDOX1 and CcDOX2 were located in subclades a and b, respectively. The proteins in subclades a and b had the N-terminal DOX domain and the C-terminal P450-related domain. The heme signature motifs (FxxGx(H/R)xCxG) of almost all of the proteins in subclades a and b were not conserved (Fig. S3). The C-terminal domains of those in subclade c consisted of proteins that harbored an apparently complete heme signature motif. Ssp1 protein is highly expressed in the teliospores of *U. maydis* (Ustilaginomycotina) (AAL38020.1) (16) and did not belong to any subclade.

### Enzymatic properties of CcDOX1 and 2

It has been reported that recombinant *A. nidulans* PpoC (AnPpoC) is unstable when the enzyme is expressed in *Escherichia coli* (13). It has also been reported that recombinant *G. graminis* linoleate diol synthase (GgLDS) expressed with *Pichia pastoris* showed a product specificity different from that observed for the enzyme prepared from the mycelia of *G. graminis* (27), while recombinant GgLDS expressed with insect cells (Sf9) showed the same product specificity (28). Accordingly, in this study, we chose insect cells as the host for expression of recombinant CcDOXs. Recombinant proteins encoded by *Ccdox1* and *2* were transiently expressed in BmN4 cells derived from the silkworm *Bombyx mori* with or without enhanced GFP (EGFP) fused at their C-terminals. To evaluate the enzymatic activity of the recombinant proteins, the cell lysate expressing the respective proteins was reacted with linoleic acid, and the products were analyzed in the negative-enhanced mass spectrum mode of LC-MS/MS. Chromatograms were drawn by extracting the molecular ions or fragment ions associated with the oxygenated products of linoleic acid, such as hydroperoxides, hydroxides, diols, or epoxyalcohols, which were expected based on the reaction of the Ascomycota Ppo enzymes (10). Accordingly, the formation of the hydroperoxide derivative of linoleic acid (*m/z* 293, [C_18_H_32_O_4_-H_3_O^+^]^−^) and the hydroxide of linoleic acid (*m/z* 295, [C_18_H_32_O_3_-H^+^]^−^) was evident in the crude lysate prepared from the insect cells expressing all four recombinant CcDOXs (CcDOX1 or CcDOX2, with/without EGFP) (Fig. S4). No sign suggestive of compounds other than hydroperoxide and hydroxide of linoleic acid was detected. Peaks 3 and 6 apparent with *m/z* 295 were tentatively assigned as 10-hydroperoxide and 8-hydroperoxide of oleic acid ([C_18_H_34_O_4_-H_3_O^+^]^−^), respectively (Fig. S5). They were not detected when purified CcDOX1/2 was reacted with linoleic acid. They were most likely formed from oleic acid endogenous to insect cells. Interestingly, the hydroperoxides of oleic acid were not found in the insect cells before disruption. This suggests that either the recombinant CcDOXs in the insect cells were in latent states and were activated upon cell disruption or that the hydrolysis of membrane lipids facilitated by cell disruption supplied free oleic acid as a substrate for the recombinant CcDOXs. Based on the chromatograms, fusion of the EGFP sequence to the C terminus of either CcDOX1 or CcDOX2 had little effect on the activity and product specificity of the reaction. The hydroxides were likely formed by unknown components derived from the cell lysates because the reduction was not observed when purified CcDOX1/2 was used for product analysis.

Recombinant CcDOX1 and CcDOX2 proteins were purified by immunoprecipitation with an anti-GFP antibody (Fig. S6). Linoleic acid was added to the purified recombinant CcDOX1 and CcDOX2, and the products were extracted, reduced with triphenylphosphine, and analyzed by LC-MS/MS in the negative enhanced product ion mode (Fig. S7). With this analysis, a peak tentatively assigned as 10-hydroxides or 8-hydroxides of linoleic acid based on the fragment ion diagnostic to the position of the hydroxide group (7) was detected as the product formed by CcDOX1 or CcDOX2, respectively.

### Expression of Ccdoxs

**Figure 4:**
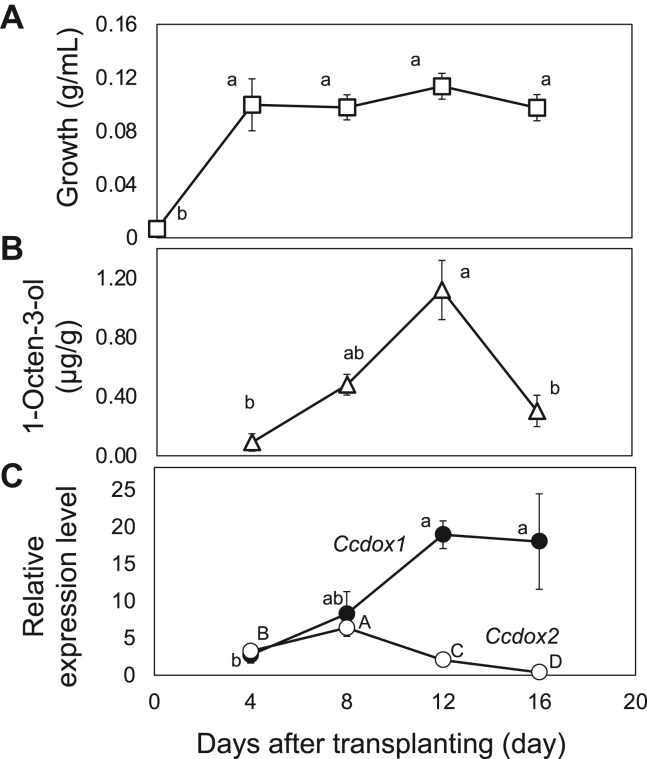
**Changes in 1-octen-3-ol forming ability and Ccdox gene expression with growth.** Developmental time course of growth (*A*), the amount of 1-octen-3-ol after freeze-thaw treatment (*B*), and expression level of *Ccdox1* and *Ccdox2* genes (*C*) in *C. cinerea* mycelia grown under a static liquid YMG media are shown. *C. cinerea* mycelia were inoculated into YMG liquid media and grown for 16 days. 1-Octen-3-ol was extracted with MTBE for GC-MS analysis. The relative expression levels of *Ccdox1* and *Ccdox2* were determined by RT-qPCR by using β-tubulin as a reference gene. The means ± SD (error bar, *n* = 3) are shown. The different letters indicate significant differences, as identified using one way ANOVA with Fisher’s least significant difference; *p* < 0.05. MTBE, methyl *tert*-butyl ether; RT-qPCR, reverse transcription–quantitative PCR; YMG, yeast extract-malt extract-glucose.

To examine the expression of *Ccdox* genes, *C. cinerea* was grown in liquid YMG medium in a static culture. The mycelia grew rapidly under the growth conditions used, and the growth reached a plateau at 4 days after inoculation (Fig. 4*A*). The amount of 1-octen-3-ol produced by freeze-thaw treatment gradually increased from day 4, it reached a peak on day 12, and then decreased thereafter (Fig. 4*B*). The transcript level of *Ccdox1* mostly followed the amount of 1-octen-3-ol but remained high even on day 16 (Fig. 4*C*). The developmental time course of *Ccdox2* expression was distinct from those of *Ccdox1* and of the 1-octen-3-ol formation ability. The expression level of *Ccdox2* reached a peak at 8 days after transplanting. Thereafter, levels decreased to almost undetectable levels on day 16.

### Properties of CcDOX1

**Figure 5:**
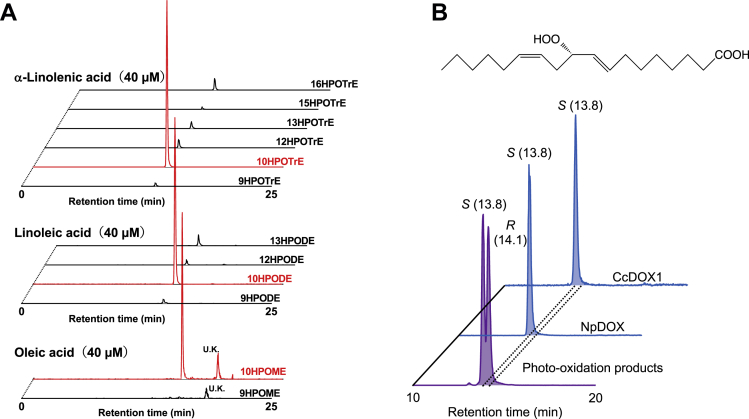
**LC-MS/MS analysis of the reaction products of recombinant CcDOX1.** Positive ionization of the hydroperoxides as their Na^+^-adducts were performed. *A*, the products from α-linolenic acid (*top*), linoleic acid (*center*), and oleic acid (*bottom*) were monitored with MRM transition specific to the hydroperoxides shown on each chromatogram. The detailed parameters for the MRM analysis are shown in Table S2. *B*, chiral phase separation of 10-hydroperoxide of linoleic acid. 10-Hydroperoxides formed by photo-oxidation reaction (*front*), by *Nostoc punctiforme* dioxygenase (NpDOX) (*center*), and by recombinant CcDOX1 (*back*) were analyzed. The structure of (8*E*,12*Z*)-10(*S*)-hydroperoxyoctadeca-8,12-dienoic acid (10(*S*)HPODE) is shown at the *top*. HPODE, hydroperoxide of linoleic acid; HPOME, hydroperoxide of oleic acid; HPOTrE, hydroperoxide of α-linolenic acid; U.K., unknown; MRM, multiple reaction monitoring.

As the expression profile of *Ccdox1* gene and the result that the main product of the recombinant CcDOX1 from linoleic acid was tentatively assigned as 10HPODE, which was reported to be the intermediate in the biosynthetic pathway to form 1-octen-3-ol in basidiomycetes, CcDOX1 was chosen for further extensive analysis. We used tandem MS analysis in the presence of sodium ions, and each isomer of the fatty acid hydroperoxide was identified and quantified using the corresponding authentic specimen (29, 30). The main product formed from linoleic acid by immunopurified recombinant CcDOX1-EGFP was confirmed to be 10HPODE (Fig. 5*A*). Other isomers, such as 9, 12, and 13HPODE, were barely detected. The positional specificity in terms of the oxygen insertion was strict, and 10-hydroperoxides were the major product, even with oleic acid or α-linolenic acid. Using chiral phase chromatography (31), the two enantiomers of 10HPODE prepared by photo-oxidation of linoleic acid were separated (Fig. 5*B*). The product formed by recombinant CcDOX1 from linoleic acid showed only one peak with the same retention time as that of 10(*S*)HPODE prepared with recombinant *Nostoc punctiforme* dioxygenase (NpDOX) (32) (Fig. 5*B*). Based on the amino acid sequence of CcDOX1 and the 3D structure of CcDOX1 predicted by AlphaFold2 (33), the binding point between the N-terminal DOX domain and the C-terminal P450 domain of CcDOX1 was inferred to be Asn670 and Pro671 (Fig. S8). When only the N-terminal DOX domain (Met1 to Asn670) of CcDOX1, excluding the C-terminal P450-related domain, was fused to EGFP and expressed in insect cells, no activity for the formation of 10HPODE from linoleic acid was detected (Fig. S9). When the enzyme activity was evaluated based on oxygen consumption, CcDOX1 showed the highest activity with linoleic acid, with a lower but still substantial activity with oleic acid and α-linolenic acid (46.6 ± 6.2 and 50.8 ± 6.5%, respectively, when compared to linoleic acid). The [S]-v plot obtained with linoleic acid was fitted to the Hill equation with the Hill coefficient (*n*) of 1.9 ± 0.27 and *K*_*A*_ value of 78 ± 9.74 μM (Fig. S10).

### Disruption of *Ccdox1*

**Figure 6:**
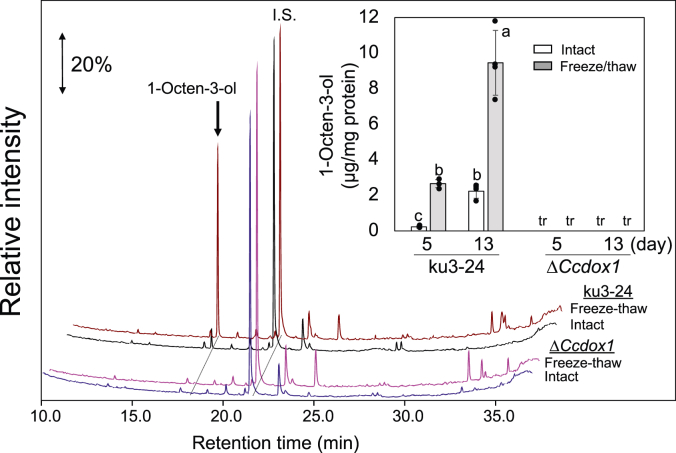
**Suppression of 1-octen-3-ol formation in the Δ*Ccdox1* strain.** Representative chromatograms obtained with intact (*blue* chromatogram) and freeze-thaw treated (*magenta*) mycelia of the Δ*Ccdox1* strain, and intact (*black*) and freeze-thaw treated (*brown*) mycelia of the parent WT strain (ku3-24). The strains were grown on YMG plate for 5 days. SPME-GC-MS techniques were employed to analyze volatiles. *Inset*, amount of 1-octen-3-ol formed with ku3-24 strain and Δ*Ccdox1* strain in intact (*white bars*) and freeze-thaw treated (*gray bars*) mycelia. The amount of 1-octen-3-ol is presented as mean ± SD (error bar, *n* = 3). The *different letters* indicate significant differences, as identified using one way ANOVA with Tukey method; *p* < 0.05. tr: trace. SPME, solid-phase microextraction; YMG, yeast extract-malt extract-glucose.

The function of *Ccdox1* was disrupted *via* homologous recombination (34) in the *C. cinerea* ku3-24 strain. Two KO lines of the *Ccdox1* gene were obtained (Fig. S11). Solid-phase microextraction (SPME)-GC/MS analysis after mycelial freeze-thaw treatment showed that the ability to form 1-octen-3-ol was highly suppressed in both KO lines (Fig. S11). Line #1 was used for further experiments. When WT *C. cinerea* (ku3-24) was cultured in YMG agar medium, 1-octen-3-ol accumulated slightly in the intact mycelia on day 5 of culture and then increased by day 13 (Fig. 6). With mycelia from both of these culture days, 1-octen-3-ol increased markedly after freeze-thaw treatment (Fig. 6). In the Δ*Ccdox1* strain, both the rapid formation of 1-octen-3-ol after freeze-thaw treatment of mycelia and the constant formation in intact mycelia were highly suppressed (Fig. 6, inset). The growth of Δ*Ccdox1* mycelia was slightly better than that of the parent line (ku3-24) (Fig. S12). Both strains set fruiting bodies with no apparent differences in morphology and timing (Fig. S12).

### 10(*S*)-hydroperoxide is cleaved into 1-octen-3-ol, independent of CcDOX1

**Figure 7:**
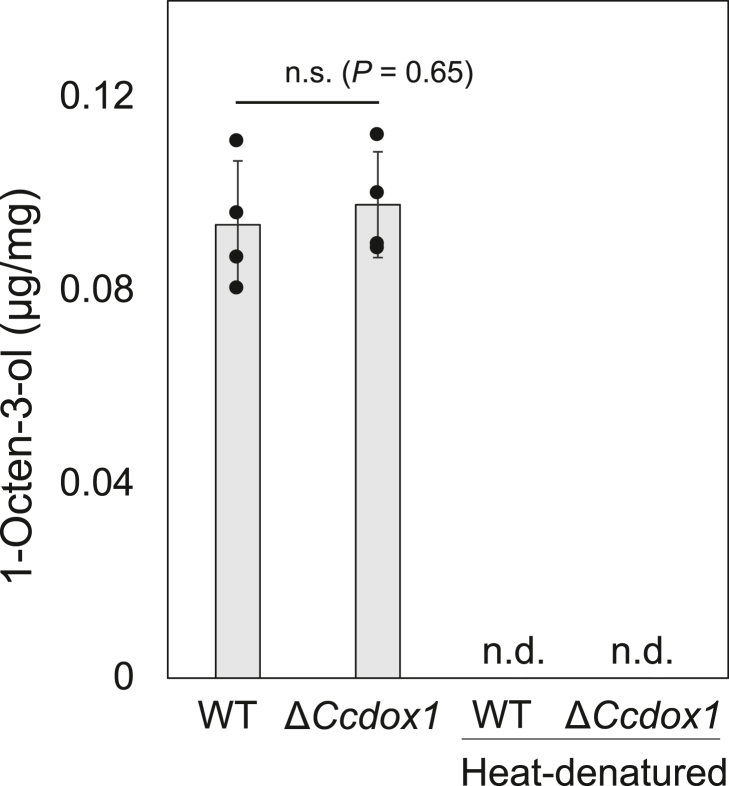
**1-Octen-3-ol formation from 10(*S*)-hydroperoxide of linoleic acid with the microsome fraction prepared from the mycelia of the WT (ku3-24) strain and Δ*Ccdox1* strain.** Reaction with heat-denatured microsome fraction formed barely any 1-octen-3-ol. Mean ± SD (*n* = 3) is shown. Student’s *t* test indicated that there was no significant difference in the amount of 1-octen-3-ol formed by WT and Δ*Ccdox1* (*p* = 0.65).

Although CcDOX1 lacks the Cys residue essential for functional cytochrome P450 activity, the possibility that CcDOX1 catalyzes the rearrangement reaction on 10(*S*)HPODE to form 1-octen-3-ol cannot be fully ruled out. This is because a few fatty acid oxygenases catalyze similar rearrangement reactions, even without any other domain that might account for the rearrangement reaction (35, 36, 37, 38). When immunopurified recombinant CcDOX1 was placed with 10(*S*)HPODE, no formation of 1-octen-3-ol was observed under the reaction conditions, while 10(*S*)HPODE was converted into 1-octen-3-ol with the microsome membrane fraction prepared from mycelia of WT *C. cinerea* (Fig. S13). When the reaction with 10(*S*)HPODE was carried out with the microsomal membrane fraction prepared from either the WT strain or the Δ*Ccdox1* strain, no significant difference was observed in their ability to form 1-octen-3-ol from 10(*S*)HPODE (Fig. 7).

### Effect of Δ*Ccdox1* on the behavior of organisms associating with the fungus

**Figure 8:**
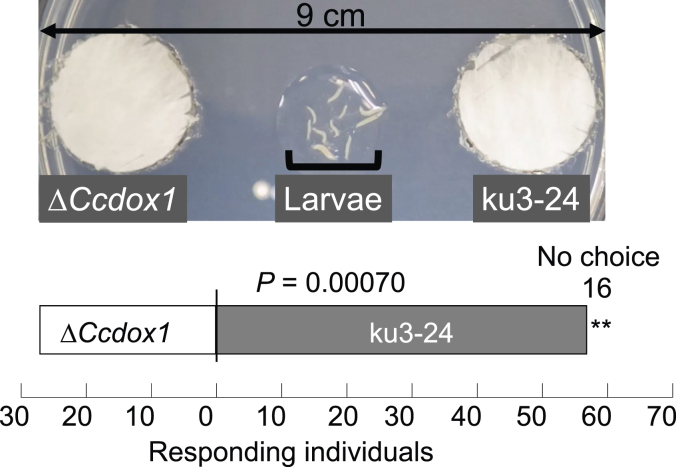
**Choice responses of *D. melanogaster* larvae to the mycelia of *C. cinerea* ku3-24 (WT) and Δ*Ccdox1*.** The arena used for the assay is shown on *top*. An assay with ten individuals was repeated ten times (total 100 individual). ∗∗*p* < 0.01 (binominal test). The experiment was repeated with the same results.

*Drosophila melanogaster* larvae show chemotaxis toward mushroom odor, especially toward 1-octen-3-ol (39). We examined the olfactory preferences of *D. melanogaster* larvae toward the mycelia of *C. cinerea* ku3-24 and Δ*Ccdox1* strains (Fig. 8). The larvae showed a significant preference for ku3-24 mycelia when compared to Δ*Ccdox1*.

*Neoempheria dilatata* belongs to a genus of fungus gnats in the family Mycetophillidae, and they can cause serious damage to shiitake mushroom cultivation in Japan (40). When the *N. dilatata* larvae were placed on the surface of the mycelia of ku3-24 or Δ*Ccdox1*, they fed on the mycelia of both strains in almost the same manner (Fig. S14). They then pupated and emerged without any distinct differences.

*Aphelenchus avenae*, *Aphelenchoides besseyi*, and *Ditylenchus destructor* are fungivorous nematodes (41, 42). We examined the population growth rates of these nematodes in the WT and Δ*Ccdox1* strains. *A. avenae* and *A. besseyi* showed no distinct differences in growth between the two *C. cinerea* genotypes (Fig. S15). However, the population growth rate of *D. destructor* was slightly, but significantly, lower with Δ*Ccdox1* than with ku3-24.

## Discussion

The biosynthetic pathway to form 1-octen-3-ol from linoleic acid through 10(*S*)HPODE was proposed in 1982 in the common mushroom (*A. bisporus*) (3, 4, 5); however, the enzymes involved in this biosynthesis have never been unequivocally identified. Assuming an enzyme similar to Ppo or LOX as a possible enzyme that could oxygenate linoleic acid to form its hydroperoxide derivatives, a search of the genome of the model basidiomycete *Coprinopsis cinerea* yielded two candidate genes, *Ccdox1* and *Ccdox2*, which showed substantial homology with filamentous ascomycete *ppo*s. No proteins with substantial similarity to LOX were detected. Through further studies on these two candidate proteins, we have provided several pieces of evidence that suggests CcDOX1 in the basidiomycete *C. cinerea* is essential for the first step in 1-octen-3-ol biosynthesis, converting linoleic acid into 10(*S*)HPODE.

The first piece of evidence was obtained using the recombinant CcDOX1 protein. Recombinant CcDOX1 is a fatty acid dioxygenase that prefers linoleic acid as the substrate to either oleic acid or α-linolenic acid and it predominantly forms 10(*S*)HPODE with high stereospecificity. The recombinant CcDOX2 was also a fatty acid dioxygenase but it barely formed 10HPODE from linoleic acid; thus, the direct involvement of CcDOX2 in 1-octen-3-ol formation in *C. cinerea* was excluded. The main product of recombinant CcDOX2 from linoleic acid was tentatively assigned as 8HODE that was likely derived from 8HPODE. 8HODE is known as an allelochemical formed by a soil-dwelling basidiomycete fungus, *Laetisaria arvalis* (43). The expression of *Ccdox1* and *Ccdox2* genes during the growth of *C. cinerea* and the comparison of the changes in the ability to form 1-octen-3-ol during growth also supported the involvement of CcDOX1 in the biosynthesis of 1-octen-3-ol. Importantly, the *S*-stereochemistry of 10HPODE formed by CcDOX1 is consistent with that of 10HPODE reported to date as an intermediate for 1-octen-3-ol formation in *A. bisporus* (common mushroom), *L. edodes* (shiitake mushroom), and *T. matsutake* (Matsutake mushroom) (5, 6, 7). The second piece of evidence was obtained by disrupting the *Ccdox1* gene in *C. cinerea*. Disruption of *Ccdox1* function resulted in almost complete suppression of the ability of *C. cinerea* mycelia to form 1-octen-3-ol. The addition of 10(*S*)HPODE into a crude enzyme solution prepared from mycelia of either the WT or Δ*Ccdox1* strains resulted in efficient conversion of the hydroperoxide derivative into 1-octen-3-ol at a similar level of efficiency. This indicates that 10(*S*)HPODE is an intermediate in the biosynthesis of 1-octen-3-ol from linoleic acid in *C. cinerea* and that the *Ccdox1* gene is required only for the conversion of linoleic acid to 10(*S*)HPODE but not for the rearrangement reaction of 10(*S*)HPODE to form 1-octen-3-ol. The latter cleavage reaction is likely to be carried out by another enzyme, independent of the function of the *Ccdox1* gene.

The involvement of Ppo or Ppo-like enzymes, especially PpoCs, in 1-octen-3-ol formation in fungi has been predicted because the main product of *Aspergillus* PpoC from linoleic acid is 10HPODE (13). Moreover, the disruption of the *ppoC*-like gene in *P. anserina* and *A. luchuensis* suppresses 1-octen-3-ol production (14, 15). However, the PpoC enzymes examined so far, that is, those from *Aspergillus fumigatus* and *A. nidulans*, produced 10(*R*)HPODE that was not used for 1-octen-3-ol formation, at least with Basidiomycota (5, 6, 7). Since the stereochemistry of 1-octen-3-ol examined so far in Ascomycota and Basidiomycota is mostly the (*R*)-isomer (5, 44), species belonging to different phyla of the same kingdom, that is, Basidiomycota and Ascomycota, may form (*R*)-1-octen-3-ol from 10HPODE with a different stereochemistry. This raises an interesting perspective on the evolution of the biosynthetic pathway of 1-octen-3-ol. However, since the gene-disruption experiments of the *ppoC*-like gene were performed only in *P. anserina* and *A. luchuensis* and the characterization of the PpoC enzymes was performed in other filamentous ascomycetes, *A. nidulans* and *A. fumigatus*, it remains unclear whether 10(*R*)HPODE is responsible for 1-octen-3-ol in ascomycetes. Genetic and biochemical studies on the same gene in the same species are necessary to obtain direct evidence to verify this possibility. In addition, it is necessary to study the orthologs of *Ccdox1* in Basidiomycota to obtain a comprehensive overview of the 1-octen-3-ol formation pathways in Basidiomycota.

Most filamentous ascomycetes contain a subset of Ppo enzymes, including PpoA, PpoB, PpoC, and PpoD. Ppos in filamentous ascomycetes have been extensively studied (11). PpoA, for example, is a DOX-P450 fusion protein involved in the biosynthesis of factors that determine the timing and balance between sexual and asexual spore development in *A. nidulans* (45). However, Ppo homologs in Basidiomycota have not been studied extensively. Among Basidiomycota, one gene from *U. maydis* (Ustilaginomycotina) and three genes from *R. solani* (Cantharellales) showing homology to ascomycete *ppo*s have been reported (16, 17), but the properties and functions of the enzymes encoded by these genes have never been studied. CcDOX1 and CcDOX2 found in this study as the proteins homologous to ascomycete Ppos also contain an N-terminal DOX domain and a C-terminal P450-related domain; however, their C-terminal P450-related domains lacked sufficient features to be recognized as members of the P450 protein family, despite substantial homology with the C-terminal P450 domain of ascomycete Ppo proteins. Remarkably, CcDOX1/2 do not have the Cys residue that is crucial for the activities of almost all P450 enzymes as the fifth heme iron ligand through a heme-thiolate bond (25). The His residue, located on the two residues upstream of the Cys residue, is essential for hydroperoxide isomerase activity in *A. nidulans* PpoA (His1004) (24). This is also absent in CcDOX1/2. Accordingly, it is highly plausible that the C-terminal Cyt P450-related domains of CcDOX1/2 do not function as P450 enzymes, as confirmed by the PpoC enzymes in Ascomycota (13). In fact, the predominant products formed from linoleic acid by recombinant CcDOX1 and CcDOX2 were 10(*S*)HPODE and 8HPODE, respectively, and there was no sign of rearrangement of the hydroperoxides that would have occurred in the presence of active P450. Interestingly, the C-terminal P450-related domains of CcDOX1/2 still retain substantial sequence signatures as P450 proteins, even though the domains do not function as P450 enzymes. The expression of only the N-terminal DOX domain of CcDOX1 in insect cells showed no oxygenation activity for linoleic acid (Fig. S9). This is unexpected but suggests that the P450-related domain plays a role in the correct folding of the N-terminal DOX domain and/or in the stabilization of structure. With the pioneering works done with AnPpoA and AnPpoC (13, 24), it was reported that AnPpoA and AnPpoC form a tetramer, and the status of heme in these hemoproteins was examined with spectral analyses. The fact that the [S]-v plot of CcDOX1 with linoleic acid did not follow the Michaelis–Menten equation but followed the Hill equation with *n* = 1.9 may be associated with cooperativity based on the subunit structure of CcDOX1. The low amounts of recombinant proteins obtained after immunopurification did not allow us to investigate their structures. Detailed kinetical and structural analyses of CcDOXs are important topics for future research that should be done after developing an efficient and prolific expression system.

The phylogenetic tree we have created showed that the DOX-P450 proteins from Ascomycota and Basidiomycota were clearly separated into two corresponding clades (Fig. 3*B*), suggesting that the DOX-P450 genes belonging to each clade diversified independently in each phylum after the divergence of Ascomycota and Basidiomycota (22). When the genome sequences of representative fungal species belonging to different classes of all the subdivisions listed in MycoCosm, namely, Cryptomycota, Microsporidia, Chytridiomycota, Blastociadiomycota, Zoopagomycota, Mucoromycota, Ascomycota, and Basidiomycota (22), were searched using the CcDOX1 sequence as the query, genes containing both the N-terminal DOX and C-terminal P450-related domains were found only in Ascomycota and Basidiomycota (Table S3). The genes found in the genome sequences of *Saitoella complicata* (Taphrinomycotina), *Rhizophagus clarus* (Glomeromycotina), *Entomortierella belijakovae* (Mortierellomycotina), *Basidiobolus meristosporus* (Entomophthoromycotina), and *Rhizoclosmatium globosum* (Chytridiomycetes) showed significant sequence homology with the DOX domain of CcDOX1 but lacked the P450-related domain. This indicates that fungi acquired DOX-like genes at the early stage of divergence after the establishment of the kingdom of fungi. Thereafter, the gene fused with the P450-related gene to form an ancestral DOX-P450 fusion gene when the Dikarya diverged. The phylogenetic tree (Fig. 3*B*) and multialignment (Fig. S3) show that there are a certain number of proteins that have apparently lost P450 enzyme activity due to the substitution of Cys and/or His in the P450-related domain with other amino acid residues in the ascomycete and basidiomycete clades. It is likely that such amino acid substitutions, which are essential for P450 enzyme activity, occurred independently in these phyla.

This study confirmed that CcDOX1 is essential for 1-octen-3-ol formation from linoleic acid, as a dioxygenase, to form 10(*S*)HPODE. The second step of the biosynthetic pathway, namely, the cleavage of 10(*S*)HPODE to form 1-octen-3-ol, is independent of CcDOX1. In the cyanobacteria *N. punctiforme*, a catalase-like heme protein catalyzes the cleavage reaction to form 1-octen-3-ol from 10(*S*)HPODE (32). Plant hydroperoxide lyase, a noncanonical cytochrome P450 enzyme classified as CYP74B, cleaves 13-hydroperoxide of linolenic acid to form (*Z*)-3-hexenal and 12-oxo-9(*Z*)-dodecenoic acid (46). We found no gene that showed a substantial similarity to the cyanobacterial catalase-like gene or that was similar to plant hydroperoxide lyases (CYP74B) in the genome of *C. cinerea*. It is still unknown which enzyme accounts for the cleavage reaction. We are currently attempting to purify the ‘hydroperoxide lyase–type’ enzyme responsible for 1-octen-3-ol formation in *C. cinerea* using classic chromatography techniques.

Identification of the gene essential for 1-octen-3-ol synthesis facilitated the examination of the ecophysiological significance of 1-octen-3-ol formation in mushrooms using the Δ*Ccdox1* strain. The current knowledge regarding the function of 1-octen-3-ol in various organisms that are potentially associated with fungi (20) suggests a role for 1-octen-3-ol in fungal defense. However, the fungivores used in the present study, the fungus gnat *N. dilatata* and the fungivorous nematode *A. avenae*, fed on the WT and 1-octen-3-ol deficient strain in the same manner. Rather, significant chemotaxis toward the WT strain, rather than the 1-octen-3-ol deficient strain, was observed in *D. melanogaster* larvae, an opportunistic fungivore. This may be because the fungivores used in this study were well adapted to the defense system employed by the fungi. Therefore, 1-octen-3-ol seems to have become a kairomone for some animals that interact with mushrooms. Therefore, the behavior of a wider array of fungivores on 1-octen-3-ol deficient *C. cinerea* must be examined to further clarify the ecophysiological role of 1-octen-3-ol. Tritrophic interactions among fungi, fungivores, and parasitoids have also been reported (47). The involvement of 1-octen-3-ol in indirect defense to recruit predators/parasitoids to fungivores that actively feed on fungi should be examined.

## Experimental procedures

### Fungi

*C. cinerea* strain ku3-24 (*A43mutB43mut pab1-1 ΔCc.ku70(Flt*^*R*^*)*/a progeny of ku70dfltF_2_#92×KF3#2) and strain 326 (*A43mutB43mut pab1-1*) were used in this study. Both strains were grown at 28 °C in the dark on YMG medium (3 g yeast extract, 3 g polypeptone, 3 g malt extract, 10 g glucose, and 20 g agar in 1 l distilled water), unless otherwise indicated. For the RT-PCR experiments, mycelia of *C. cinerea* strain 326 were inoculated into YMG liquid media (10 ml in a 100 ml flask) and cultured under static conditions at 28 °C in the dark.

### Determination of volatiles

*C. cinerea* (ku3-24) was grown on YMG agar for 5 days, after which 0.5 cm^3^ mycelia with the YMG agar were cut out and sealed in a glass vial (20 ml). Mycelia were subsequently frozen in liquid nitrogen for 2 min and thawed in a water bath at 35 °C for 10 min to disrupt the tissues. The volatiles formed were extracted from the headspace using an SPME fiber (50/30 μm DVB/CAR/PDMS, Merck & Co) at 30 °C for 30 min. SPME-GC-MS was performed on a QP-5050 (Shimadzu) instrument equipped with a 0.25 mm × 30 m DB-WAX column (film thickness 0.25 μm; Restek). The SPME fiber was inserted into the injection port set at 200 °C for 10 min in the splitless mode. The sampling time was 1 min. The column temperature was initially set at 40 °C for 5 min, increased by 5 °C min^−1^ to 200 °C, and then maintained at 200 °C for 2 min. The carrier gas (He) was supplied at a flow rate of 86.1 kPa. The injector and interface temperatures were maintained at 200 °C. The mass detector was operated in the electron impact mode with an ionization energy of 70 eV. 1-Octen-3-ol was assigned by comparing the MS profile and retention time of authentic 1-octen-3-ol (Tokyo Chemical Industry Co, Ltd) and quantified using an externally constructed calibration curve. When required, the fragment ion (*m/z* 72) was monitored to detect 1-octen-3-ol with high specificity. 3-Methyl-1-butanol, 3-octanone and 1-octanol were identified by analyzing the MS profiles using the NIST08s database.

The volatiles were extracted with methyl *tert*-butyl ether (MTBE) and analyzed. After the freeze-thaw treatment, 1 ml of MTBE containing *n*-nonanyl acetate (internal standard, 1 μg ml^−1^) was added, and the mixture was centrifuged at 21,500*g* for 10 min to collect the upper organic phase. The protein content was determined using a protein assay kit (Bio-Rad). To examine the effect of reagents on the ability to form 1-octen-3-ol (Fig. 2, *C* and *D*), mycelia grown on YMG agar were placed into a 2 ml microtube with 0.5 g glass beads (420–500 μm i.d.) and 400 μl of 50 mM Tris–HCl (pH 8.0) containing the reagent of interest. Subsequently, the mycelia were homogenized using a bead-homogenizer MS-100 (TOMY) at 3500 rpm for 1 min. The volatiles formed were extracted by adding 1 ml MTBE containing the internal standard for GC-MS analysis. To examine the developmental changes in the ability to form 1-octen-3-ol in the mycelia grown in liquid culture, mycelia (strain 326) were collected every 4 days, washed with distilled water, and finally weighed. A portion of the mycelia was freeze-thaw treated (2 min in liquid N_2_ and subsequently 10 min at 35 °C) in a closed glass tube. 1-Octen-3-ol formed was extracted with MTBE containing an internal standard and subjected to GC-MS analysis, as described previously.

### Phylogenetic analysis

Phylogenetic analysis was performed using the maximum likelihood method based on the LG model + G + I in MEGA X. Amino acid sequences were aligned using MAFFT v7.475 and Gblocks Server v0.91b. GenBank accession numbers of each protein used for phylogenetic analysis are listed in Table S1.

### Recombinant protein

Total RNA was extracted from the mycelium of *C. cinerea* strain 326 using the RNeasy Plant Mini kit (Qiagen) and treated with a DNA-free kit (Thermo Fisher Scientific). Complementary DNA (cDNA) was synthesized using the ReverTra Ace quantitative PCR (qPCR) RT Master Mix (TOYOBO). The coding regions of *Ccdox1* and *Ccdox2* cDNA were amplified by PCR using PrimeSTAR Max DNA Polymerase (Takara Bio Inc) along with the primers listed in Table S4 and were subcloned into the pA3hr5 vector (provided by Professor Kobayashi at Yamaguchi University) using the In-Fusion HD Cloning kit (Takara). Recombinant CcDOX proteins, with and without EGFP, were prepared. When only the N-terminal DOX domain of CcDOX1 was expressed, the 3D structural model of CcDOX1 predicted with AlphaFold2 was examined (Fig. S8). Based on the model structure, the junction between the N-terminal and C-terminal P450 domains of CcDOX1 was assigned as the site between Asn670 and Pro671. Accordingly, a protein sequence ranging from the initial Met to Asn670 was expressed as an EGFP-fusion protein. PEI (4 μg) (Techno Chemical Co) in 100 μl of WakoVAC PSFM-J1 medium (serum-free medium; FUJIFILM Wako Pure Chemical Co) was gently mixed with 2 μg of cDNA in the pA3hr5 vector dissolved in 100 μl of serum-free medium by pipetting, and the mixture was incubated at 25 °C for 15 min. BmN4 cells (from *B. mori*) were poured into a plate (40 mm × 13.5 mm) and allowed to stand for at least 15 min to adhere to the bottom surface, after which the medium was carefully removed to prevent detachment. The plasmid DNA mixture was diluted with 800 μl serum-free medium and poured onto BmN4 cells on the plate. The plate was then sealed and incubated at 28 °C in the dark for 4 days. The cells were harvested by centrifugation at 400*g* for 10 min and washed with lysis buffer (50 mM potassium phosphate buffer (pH 7.4), 1 mM EDTA, 1 mM glutathione, 5% glycerol, 0.04% Tween 20). They were then suspended in 600 μl of lysis buffer and sonicated with an Ultrasonic Disrupter UD-201 (TOMY) three times with 5 s pulses while on ice. The cell-free lysate was collected after centrifugation at 15,000*g* for 30 min at 4 °C. In some experiments, the crude cell lysate supernatant was used as the enzyme source. The expressed protein fused with EGFP was immunoprecipitated with 10 μl Dynabead Protein G (Thermo Fisher Scientific) and 2 μg of anti-GFP mAb (Fujifilm Wako Pure Chemicals). The beads were washed twice with lysis buffer and suspended in 147 μl lysis buffer for enzyme assay and SDS-PAGE analysis.

### Enzyme assay and product analysis

Recombinant CcDOX1 fused with EGFP was incubated with 10 to 500 μM linoleic acid, oleic acid, or α-linolenic acid for 30 min on ice. The reaction was terminated by adding three volumes of ethanol. After centrifugation at 15,000*g* for 30 min at 4 °C, the supernatant was filtered using a DISMIC-03JP (0.50 μm; ADVANTEC). The clear supernatant was dried under N_2_ gas flow, and the residue was dissolved in methanol containing 0.1 mM sodium acetate. The oxygenated fatty acids were analyzed with an HPLC-MS/MS system comprising a 3200 QTRAP (SCIEX) using an enhanced MS scan mode with negative electrospray ionization (ion spray voltage, −4500 V at 300 °C; using nitrogen as both the curtain gas (set to 40 arbitrary units) and collision gas (set to “high”); collision energy, −10 V; declustering potential, −10 V). The products were separated on a Mightysil RP18 column (5 μm, 2 mm inner diameter × 150 mm, Kanto Chemical) using solvent A (water/formic acid, 100:0.1, v/v) and solvent B (acetonitrile/formic acid, 100:0.1, v/v). Elution conditions were as follows: 0 to 5.0 min, 20% B; 5.0 to 20.0 min, 20% to 60% B; 20.0 to 50.0 min, 60% to 65% B; 50.0 to 60.0 min, 65% to 100% B and 60.0 to 70.0 min, 100% B, with a flow rate of 0.2 ml min^−1^. The [S]-v plot of recombinant CcDOX1 with linoleic acid was constructed by analyzing the peak area obtained by LC-MS/MS analysis in the negative ion mode. The largest peak obtained with 500 μM of linoleic acid was set to 100%. The data obtained were analyzed with Origin software (OriginPro 2022, OriginLab Co). To gain insight into the positional specificity of the oxygenation reaction, the hydroperoxides were reduced to the corresponding hydroxides by reacting with triphenylphosphine, and they were subjected to HPLC-MS/MS analysis with enhanced product ion mode with a negative ion of *m/z* 295.30 or *m/z* 297.20, corresponding to the hydroxide of linoleic acid (C_18_H_32_O_3_-H^+^) or hydroxide of oleic acid (C_18_H_34_O_3_-H^+^), as the parent ion. Elution conditions were as follows: 0 to 5.0 min, 20% B; 5.0 to 30.0 min, 20% to 60% B; 30.0 to 100.0 min, 60% to 65% B; 100.0 to 120.0 min, 65% to 100% B and 120.0 to 130.0 min, 100% B, with a flow rate of 0.2 ml min^−1^. The fragment ion of *m/z* 183.1 or *m/z* 157.1 formed with a collision energy of −30 V, which followed the criteria for 10-hydroxide or 8-hydroxide of linoleic acid, respectively (48). 10-Hydroxide or 8-hydroxide of oleic acid was tentatively assigned with the fragment ion of *m/z* 155.2 or *m/z* 157.1, respectively.

For complete structural determination and quantification, the HPLC-MS/MS system (4000 QTRAP quadrupole/linear ion trap tandem mass spectrometer (SCIEX)) in multiple reaction monitoring mode with positive electrospray ionization after the addition of Na^+^ ions was used (29). An Inertsil ODS-3 column (5 μm, 2.1 mm × 150 mm, GL Sciences) was used with a column temperature of 40 °C. The mobile phases used were water/formic acid (99.9:0.1, v/v, solvent A) and methanol/formic acid (99.9:0.1, v/v, solvent B). Elution conditions were as follows: 0 to 5.0 min, 60% B; 5.0 to 15.0 min, 60% to 100% B; and 15.0 to 20.0 min, 100% B. The flow rate was maintained as follows: 0 to 5.0 min, 0.3 ml min^−1^; 5.0 to 20.0 min, 0.2 ml min^−1^, 20.0 to 25.0 min, 0.3 ml min^−1^. The injection volume was 10 μl. The transitions used to identify each fatty acid hydroperoxide are listed in Table S2 (30). The column eluent was mixed with methanol containing 2 mM sodium acetate, which was added at 0.01 ml min^−1^. Each isomer of the fatty acid hydroperoxide was identified by the retention time and MS/MS profile of its Na^+^-adduct using a standard compound (30). For the separation of the enantiomers (31), a CHIRALPAK ID (2.1 mm i.d. × 150 mm, 5 μm, Daicel) column was used with a column temperature of 40 °C. Water/formic acid (99.9:0.1, v/v, solvent A) and methanol/formic acid (99.9:0.1, v/v, solvent B) were used as the mobile phases, and the elution conditions were as follows: 0 to 2.0 min, 60% B; 2.0 to 15.0 min, 60% to 90% B; 15.0 to 18.0 min, 90% B. The flow rate was 0.2 ml min^−1^. The column eluent was mixed with methanol containing 2 mM sodium acetate, which was added at 0.01 ml min^−1^.

The 10-hydroperoxide of linoleic acid was prepared using recombinant NpDOX protein expressed in *E. coli* cells. The codon usage of *N. punctiforme* PCC73102 animal heme peroxidase (NpDOX) gene (GenBank: CP001037.1) was optimized for *E. coli*, the ORF was subcloned into the pET15b vector using the In-Fusion HD Cloning Kit (Takara Bio), and *E. coli* BL21 Star (DE3) (Thermo Fisher Scientific) cells were transformed with the plasmid. The cells were grown in LB medium containing 100 μg ml^−1^ ampicillin at 37 °C to an optical density at 600 nm of 0.6 to 0.8 (Shimadzu, UV-1900i). The expression of the recombinant protein was induced by adding IPTG to a final concentration of 0.1 mM. After cultivation at 16 °C for 18 h, cells were harvested by centrifugation at 10,000*g* for 10 min. They were then suspended in 5 ml of 50 mM Tris–HCl buffer (pH 8.75) and lysed by sonication with an Ultrasonic Disrupter UD-201 (TOMY) three times with 10 s pulses on ice. The cell lysate was centrifuged at 10,000*g* for 30 min at 4 °C and the supernatant was collected. The supernatant was incubated with 5 mM of linoleic acid for 30 min on ice. The reaction mixture was acidified by adding diluted HCl and the product was extracted with ethyl acetate (3 ml). 10(*S*)HPODE was purified using preparative TLC.

To determine the substrate specificity, dioxygenase activity was monitored using a Clark-type oxygen electrode (Rank Brothers Ltd) at 25 °C. The reaction was initiated by the addition of 6 μl linoleic acid (18:2), oleic acid (18:1), or α-linolenic acid (18:3) at 50 mM in 294 μl of lysis buffer containing purified CcDOX1 and stirred with a magnetic stirrer.

### Expression of *Ccdoxs*

The mycelia of *C. cinerea* were inoculated into YMG liquid medium (10 ml in a 100 ml flask) and cultured under static conditions at 28 °C. Mycelia were collected every 4 days, washed with distilled water, and weighed. RNA was extracted from the mycelia using the RNeasy Plant Mini Kit (Qiagen) and treated with a DNA-free kit (Thermo Fisher). cDNA was constructed using ReverTra Ace RT Master Mix (Toyobo), and reverse transcription-qPCR was performed using SYBR Green (Kapa Biosystems). β-Tubulin (*CC1G_04743*) was used as the reference gene in the experiment. The primers used for reverse transcription-qPCR are listed in Table S4.

### Disruption of *Ccdox1*

A genomic fragment containing *Ccdox1*, amplified using genomic DNA from *C. cinerea* ku3-24 as a template, and the primer set SV13-SV14 (Table S4) was cloned into pBluescript II KS+ digested with EcoRV. Inverse PCR was performed with the resulting plasmid as a template and the primer set SV17-SV18. A DNA fragment containing the expression cassette for hygromycin resistance was also amplified by PCR using pTN2005 as the template and the primer set M13F-M13R. The two resulting DNA fragments were fused using the GeneArt Seamless Cloning and Assembly Kit (Thermo Fisher) to yield a plasmid containing the Ccdox1-disruption cassette (34). Transformation was performed as described by Nakazawa *et al.* (49) using protoplasts prepared from mycelial cells.

### Cleavage reaction

The mycelia of *C. cinerea* strains ku3-24 and Δ*Ccdox1* were cultivated at 28 °C for 12 days in YMG liquid medium. Mycelia were collected and washed with 100 mM Tris–HCl (pH 7.5). Mycelia (5 g) were suspended in 10 ml of 100 mM Tris–HCl (pH 7.5) and homogenized using a mortar and pestle on ice. The homogenate was centrifuged at 15,000*g* for 30 min at 4 °C and then the supernatant was subsequently centrifuged at 138,000*g* for 60 min at 4 °C. The collected microsome fraction was suspended in 400 μl of 100 mM Tris–HCl (pH 7.5). Microsomes were incubated with 1.5 μM 10(*S*)HPODE at 25 °C for 10 min. The products were extracted with 200 μl of MTBE containing the internal standard for GC-MS analysis. Thermal denaturation was performed at 90 °C for 10 min.

### Fungivores

The WT strain (Oregon R) of the fruit fly *D. melanogaster* was reared at 24 °C. *C. cinerea* ku3-24 and Δ*Ccdox1* strains were grown on YMG agar plates (90 mm diameter) until their mycelia covered the entire surface of the plate. Agar plugs with a diameter of 20 mm were removed from the middle of the region between the center and periphery of the plates using a cork borer and inserted into holes made at the periphery of the 90 mm chemotaxis plates (17 g agar, 1 ml 1 M CaCl_2_, 1 ml 1 M MgSO_4_, 5 ml 1 M KH_2_PO_4_, pH 6.0, in 1 l water) (39). Ten third-instar larvae of *D. melanogaster* were placed at the center of the arena, and the number of larvae that reached the respective mycelia within 10 min was counted as those attracted to the agar plug. Agar plugs from plain YMG plates were used as the controls.

Individual larvae (7–10 mm) of the fungus gnat *N. dilatata* (40), reared on mycelial blocks of shiitake mushrooms (*L. edodes*) (Mori Co), were placed on the surface of the mycelia of *C. cinerea* that fully covered the surface of YMG plates. The plate was kept for 24 h in the dark at 25 °C, and the area consumed by the fungivore was measured using the ImageJ software (https://imagej.nih.gov/ij/).

The fungal-feeding nematodes, *A. avenae*, *A. besseyi*, and *D. destructor*, were maintained on a *Botrytis cinerea* strain that lacks the ability to form spores as a food fungus. The strain was kept on a 5-fold diluted (1/5 ×) potato dextrose agar (PDA) plate (Eiken Chemical Co) (41). Nematode propagation was compared using a 1/5 × PDA plate (9 cm diameter) seeded with the respective strains of *C. cinerea*. A 0.6 cm^2^ piece of 1/5 × PDA with mycelia was inoculated on a fresh 1/5 × PDA plate and incubated at 20 °C for 2 weeks. Thereafter, 200 mixed-stage nematodes were inoculated on the plates. Four weeks after inoculation, nematodes were recovered from the plates using the Baermann method (41). After repeated washing of the harvested nematodes with distilled water, protein content was determined using a bicinchoninic acid protein assay kit (Thermo Fisher Scientific). The population growth rate was calculated by dividing the protein content obtained after 4 weeks by the initial protein content of 200 nematode individuals.

## Data availability

The data that support the findings of this study are available from the corresponding author upon reasonable request.

## Supporting information

This article contains supporting information (22, 29).

## Conflict of interest

The authors declare that they have no conflicts of interest with the contents of this article.
