# Supplementary material for: Coprinopsis cinerea dioxygenase is an oxygenase forming 10(S)-hydroperoxide of linoleic acid, essential for mushroom alcohol, 1-octen-3-ol, synthesis
Source: J Biol Chem. 2022 Sep 17;298(11):102507. doi: 10.1016/j.jbc.2022.102507 (PMC9579011; doi:10.1016/j.jbc.2022.102507)

Supplementary Information for

*Coprinopsis cinerea* dioxygenase is an oxygenase forming 10(*S*)-hydroperoxide of linoleic acid, essential for formation of mushroom alcohol, 1-octen-3-ol

Takuya Teshima, Risa Funai, Takehito Nakazawa, Junya Ito, Toshihiko Utsumi, Pattana Kakumyan, Hiromi Mukai, Toyoshi Yoshiga, Ryutaro Murakami, Kiyotaka Nakagawa, Yoichi Honda, Kenji Matsui*

*Kenji Matsui

Email: [matsui@yamaguchi-u.ac.jp](mailto:matsui@yamaguchi-u.ac.jp)

This PDF file includes:

Figures S1 to S15

Tables S1 to S4

**Fig. S1**. Alignment of the N-terminal DOX domains of *A. nidulans* AnPpoA (GenBank; AAR88626.1), AnPpoC (AAT36614.1), *C. cinerea* CcDOX1 (EAU90460.2), CcDOX2 (EAU86789.2), *Agaricus bisporus* AbDOX1 (EKV46570.1), AbDOX2 (EKV43127.1), and mouse (*Mus musculus*) MmCOX2 (Q05769.1). The amino acid residues described in the text are indicated by colored arrows with numbering based on the CcDOX1 (blue background) and MmCOX2 (red background) sequences, respectively.

**Fig. S2**. Alignment of the C-terminal cytochrome P450 domains of AnPpoA, AnPpoC, CcDOX1, CcDOX2, AbDOX1, AbDOX2, and *Fusarium oxysporum* CYP450nor (P23295.2). The ExxR motif widely conserved in P450 enzymes and the heme signature motif (FxxGxHxCxG) essential for P450 activities are highlighted.

**Fig. S3.** Alignment of the heme signature motif (FxxGxHxCxG) from the protein sequences used for the construction of the phylogenetic tree shown in Fig. 3B.

**
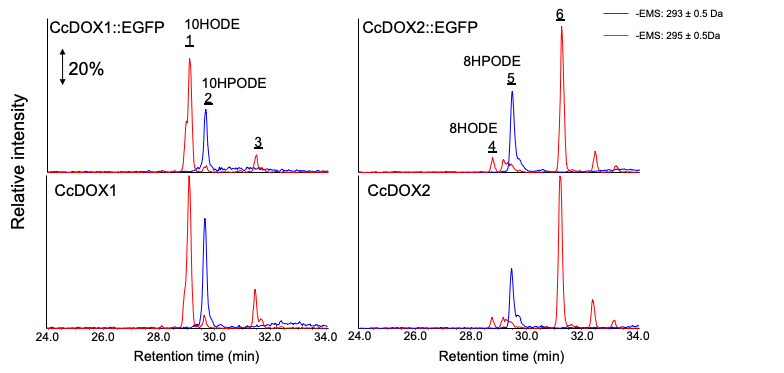
**

**Fig. S4.** Products formed by insect cell-expressed recombinant CcDOX1 and CcDOX2 with/without EGFP. The crude lysate of the insect cells expressing CcDOX1, CcDOX1 fused with EGFP (CcDOX1::EGFP), CcDOX2, and CcDOX2 fused with EGFP (CcDOX2::EGFP) had linoleic acid added to them, and the products were analyzed in the negative enhanced mass spectrum mode of LC-MS/MS. Chromatograms of extracted ions of *m/z* 293.0 ± 0.5 corresponding to linoleic acid hydroperoxide [M-H_3_O^+^]^-^ and *m/z* 295.0 ± 0.5 corresponding to linoleic acid hydroxide [M-H^+^]^-^ are shown in blue and red, respectively. Peak 1 and 2 were tentatively assigned as 10HODE and 10HPODE, respectively. Peak 4 and 5 were tentatively assigned as 8HODE and 8HPODE, respectively. The conversion of hydroperoxide into hydroxide was likely catalyzed by an unknown enzyme in the insect cells. Peak 3 and 6 were likely derived from oleic acid endogenous to insect cells.


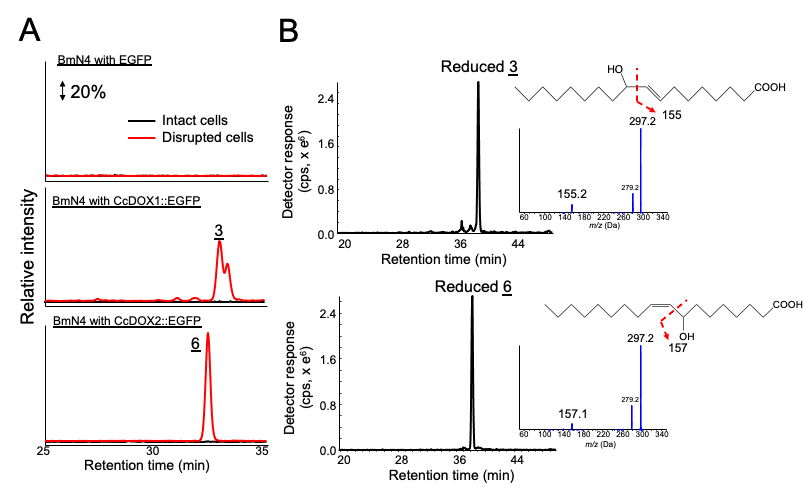


Fig. S5. Products formed by the insect cells (BmN4) expressing EGFP, CcDOX1::EGFP, or CcDOX2::EGFP. (A) The intact cells (black line) or the cells after disruption with a sonicator (red line) were extracted with ethanol for LC-MS/MS analysis in the negative enhanced mass spectrum. Chromatograms of extracted ions of *m/z* 295.0 ± 0.5 corresponding to oleic acid hydroperoxide [C_18_H_34_O_4_-H_3_O^+^]^-^ are shown. (B) The products formed in the disrupted insect cells containing recombinant CcDOX1 (upper) and CcDOX2 (lower) were reduced with triphenylphosphine, and served for LC-MS/MS analysis in the enhanced product ion mode with *m/z* 297.2 [C_18_H_34_O_3_-H^+^]^-^ as the parent ion. Based on the fragment profiles, reduced 3 and reduced 6 were tentatively assigned as 10- and 8-hydroxide of oleic acid, respectively.

 **Fig. S6.** Expression of recombinant CcDOX1 and CcDOX2 in BmN4 cells. (A) Immunoblot analysis with crude extract expressing EGFP (shown with an asterisk in lane 1, ca. 27 kDa), CcDOX1::EGFP fusion protein (lane 2), and CcDOX2::EGFP fusion protein (lane 3). The arrow indicates the protein bands corresponding to the fusion proteins. (B) CBB-staining of the crude extract expressing EGFP (lane 2), CcDOX1::EGFP fusion protein (lane 3), CcDOX2::EGFP fusion protein (lane 4), immunoprecipitated fraction of the lysate expressing EGFP only (lane 5), immune-purified CcDOX1::EGFP fusion protein (lane 6), and immune-purified CcDOX2::EGFP fusion protein (lane 7). The positions of the fusion protein (147 kDa) and the heavy chain of rabbit immunoglobulin (ca. 50 kDa) used for purification are shown with an arrow and a triangle, respectively. The asterisk in the lane 5 indicates the protein bands corresponding to EGFP. Lane 1: molecular weight marker.


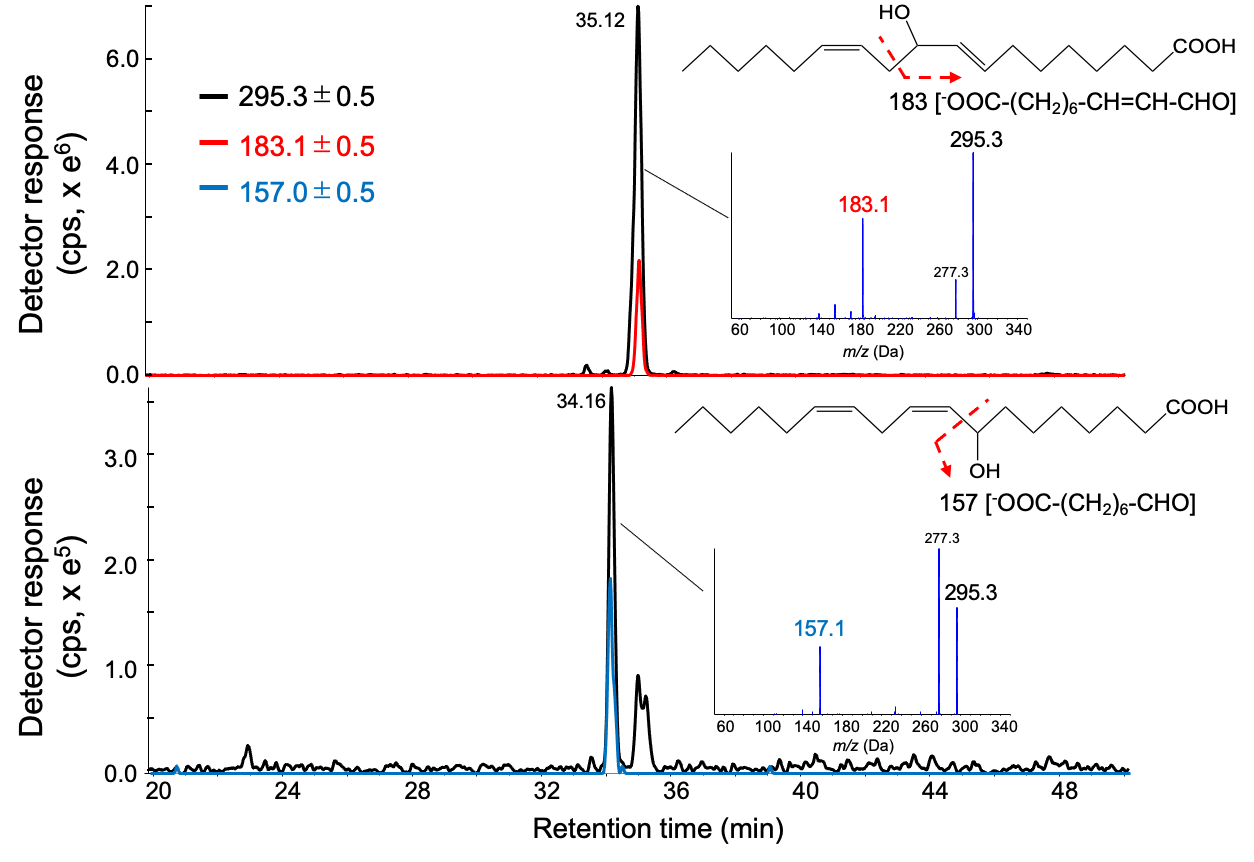


**Fig. S7**. Chromatograms with the products formed by immuno-purified recombinant CcDOX1::EGFP (upper) and immuno-purified recombinant CcDOX2::EGFP (lower) from linoleic acid. The chromatograms were obtained with LC-MS/MS in the negative enhanced product ion mode. The products were reduced with triphenylphosphine before analysis. The negative ion of *m/z* 295.30 corresponding to the hydroxides of linoleic acid [M-H^+^]^-^ was chosen as the parent ion. The black line is shown with the parent ion, and the red and blue lines are drawn with *m/z* 183.1 and *m/z* 157.1, corresponding the fragment ions diagnostic to 10- and 8-hydroxides of linoleic acid. The mass spectrum for each main peak is shown in the inset with expected fragmentation pattern.


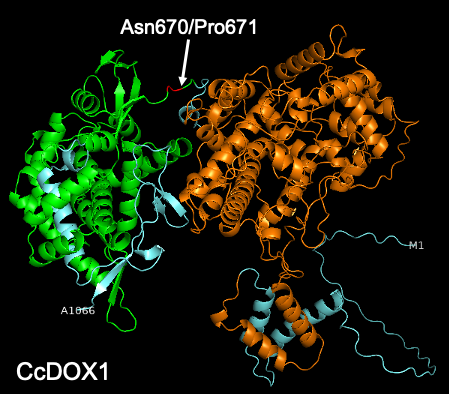


**Fig. S8.** The 3D structure of CcDOX1 predicted with AlphaFold2. The N-terminal DOX domains are shown in orange, the C-terminal P450-related domains in green, and the other regions in cyan. The predicted junction between the DOX domains and the P450-related domains are shown with a white arrow.

**Fig. S9**. Deletion of the C-terminal P450-related domain from CcDOX1 resulted in loss of activity. (A) Immunoblot analysis with the immune-purified EGFP (shown with an asterisk in lane 1, ca. 27 kDa), CcDOX1::EGFP fusion protein (lane 2), and CcDOX1 without P450-related domain::EGFP fusion protein (lane 3). The arrow indicates the protein bands corresponding to the fusion proteins. (B) Chromatograms with the products formed by immuno-purified recombinant CcDOX1::EGFP (upper) and immuno-purified recombinant CcDOX1 without P450-related domain (CcDOX1(-P450)::EGFP (lower) from linoleic acid. The chromatograms were obtained with LC-MS/MS in the negative enhanced product ion mode. The products were reduced with triphenylphosphine before analysis. The negative ion of *m/z* 295.30 corresponding to the hydroxides of linoleic acid [M-H^+^]^-^ was chosen as the parent ion. The black line is shown with the parent ion, and the blue, red, and green lines are drawn with *m/z* 183.1, *m/z* 171.1, and *m/z* 195.1, corresponding the fragment ions diagnostic to 10-, 9-, and 13-hydroxides of linoleic acid.

**Fig. S10.** [S]-v plot of recombinant CcDOX1 with linoleic acid. The relative amount of product obtained with LC-MS/MS with negative enhanced MS mode was fitted to the Hill equation (*n* = 1.86 ± 0.27) with correlation coefficient (r^2^) of 0.9899 by using Origin software. The value obtained with 1000 µM was set at 100%. *K_A_* is the ligand concentration producing half occupation.

**Fig. S11**. Disruption of *Ccdox1* gene. (A) Schematic diagram of gene disruption through homologous recombination. Primers used for the PCR reactions are shown. (B) PCR analysis to confirm the deletion of *Ccdox1* using genomic DNA as template. The primer sets used in this study are shown in Table S4. (C) Amount of 1-octen-3-ol formed by the ku3-24 and two Δ*Ccdox1* strains.


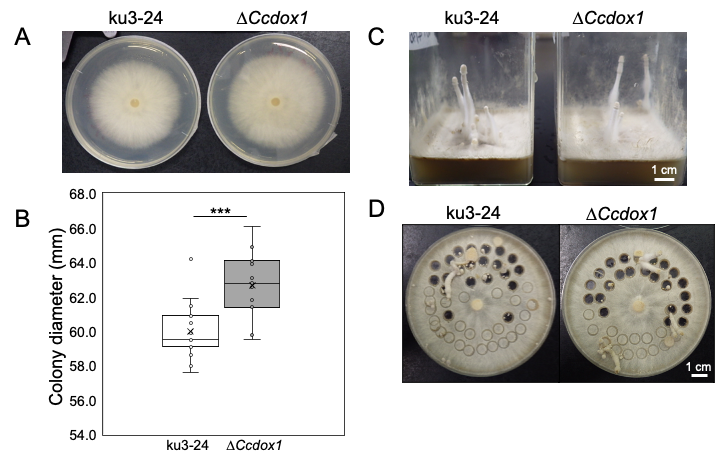


**Fig. S12**. Growth phenotypes of the wildtype and Δ*Ccdox1* mycelia grown on YMG medium. (A) Colonies formed on the 5^th^ day after the onset of culture on YMG medium. (B) Diameters of mycelial colonies measured on the 5^th^ day are presented in the respective boxplots. Statistically significant differences between ku3-24 and Δ*Ccdox1* were determined by Student’s t-test. ***P < 0.001; *n* = 15. (C) Fruiting bodies formed on the 32^nd^ day after the onset of culture on YMG medium. (D) Fruiting bodies formed on the 11^th^ day after cutting the mycelia grown for 9 days.

**Fig. S13.** The formation of 1-octen-3-ol from 10(*S*)HPODE with microsome fraction prepared with the mycelia of *C. cinerea* (red trace) and with immune-purified CcDOX1 expressed in insect cells (black). As it was anticipated that 10(*S*)HPODE spontaneously degraded to yield 1-octen-3-ol, the microsomal fraction was heat-denatured and used as the enzyme source (blue). The molecular ion chromatogram with the fragment ion of *m/z* 72 that is specific to 1-octen-3-ol is shown.


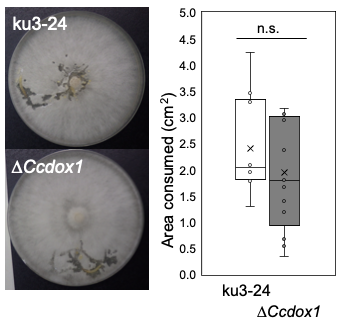


**Fig. S14.** Feeding behavior (left) of *Neoempheria dilatata* on Δ*Ccdox1* and its parent strain (ku3-24) on YMG medium. The area consumed by the larvae (right) after 24 h was measured with ImageJ. There was no statistically significant difference between the two genotypes (*n* =10, Student’s *t*-test).


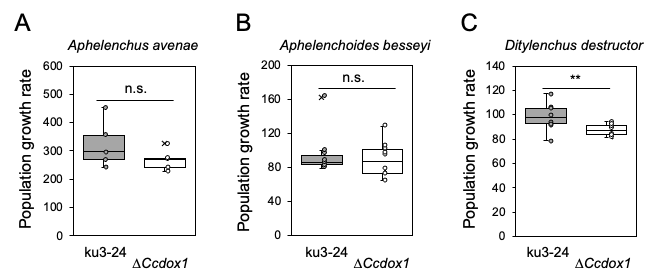


**Fig. S15**. Nematode propagation on *Coprinopsis cinerea* Δ*Ccdox1* and its parent strain ku3-24. (A) *Aphelenchus avenae*, (B) *Aphelenchoides besseyi*, and (C) *Ditylenchus destructor*. Data from 5 (*A. avenae*) and 10 (*A. besseyi* and *D. destructor*) independent experiments are presented in the respective boxplots. Statistically significant differences between ku3-24 and Δ*Ccdox1* were determined by Student’s *t*-test. ***P* < 0.01.

Table S1. Proteins used to construct the phylogenetic tree shown in Fig. 3B.


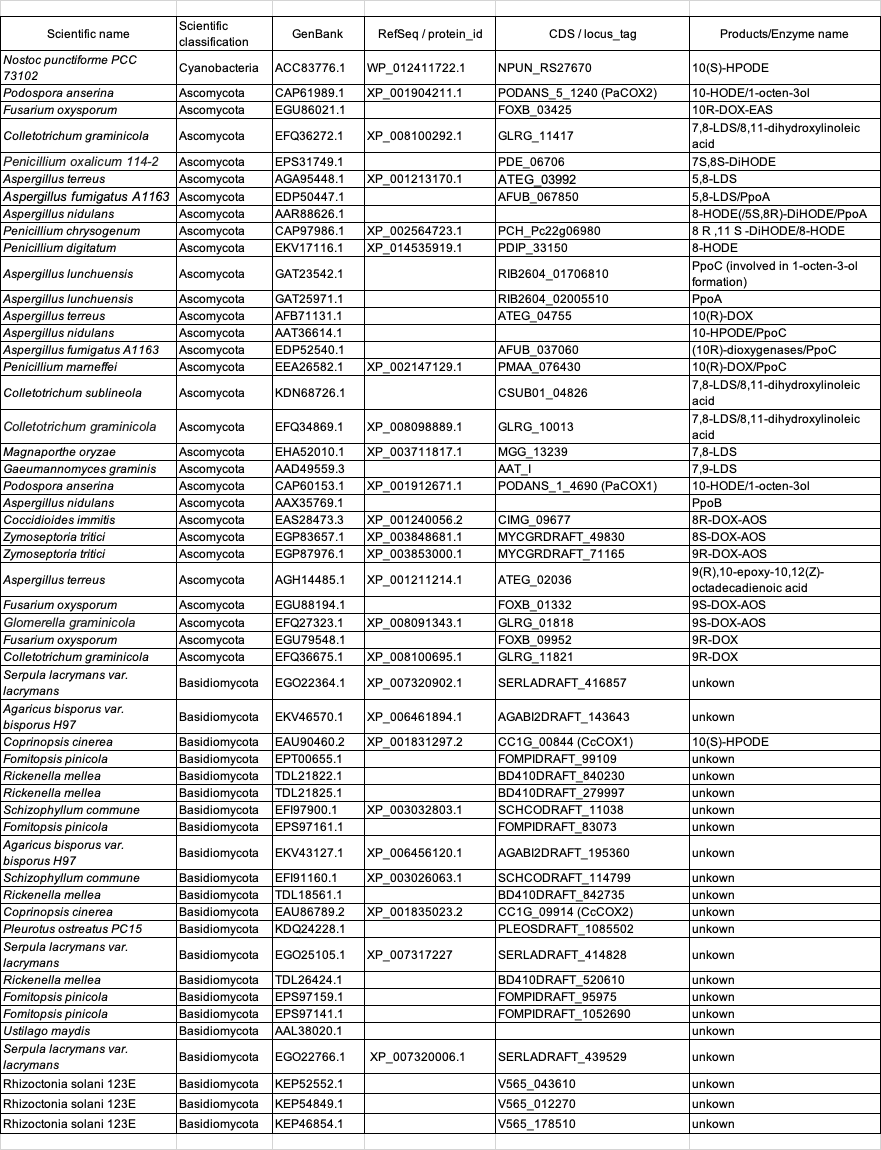


Table S2. Condition for multiple reaction monitoring to identify each hydroperoxide of fatty acid.


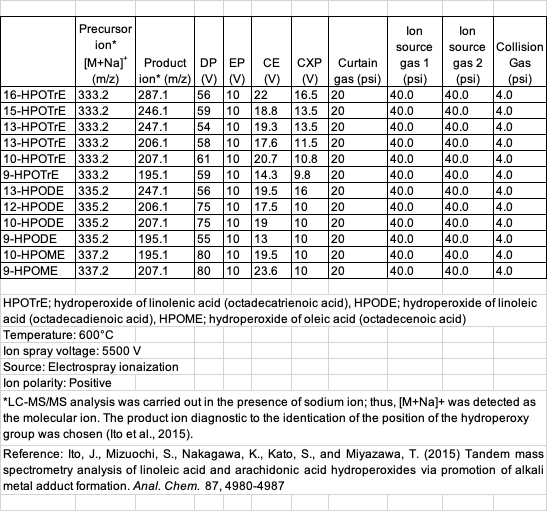


Table S3. The domain structures of the proteins showing the highest homology to CcCOX1 within the respective class in the kingdom of fungi.


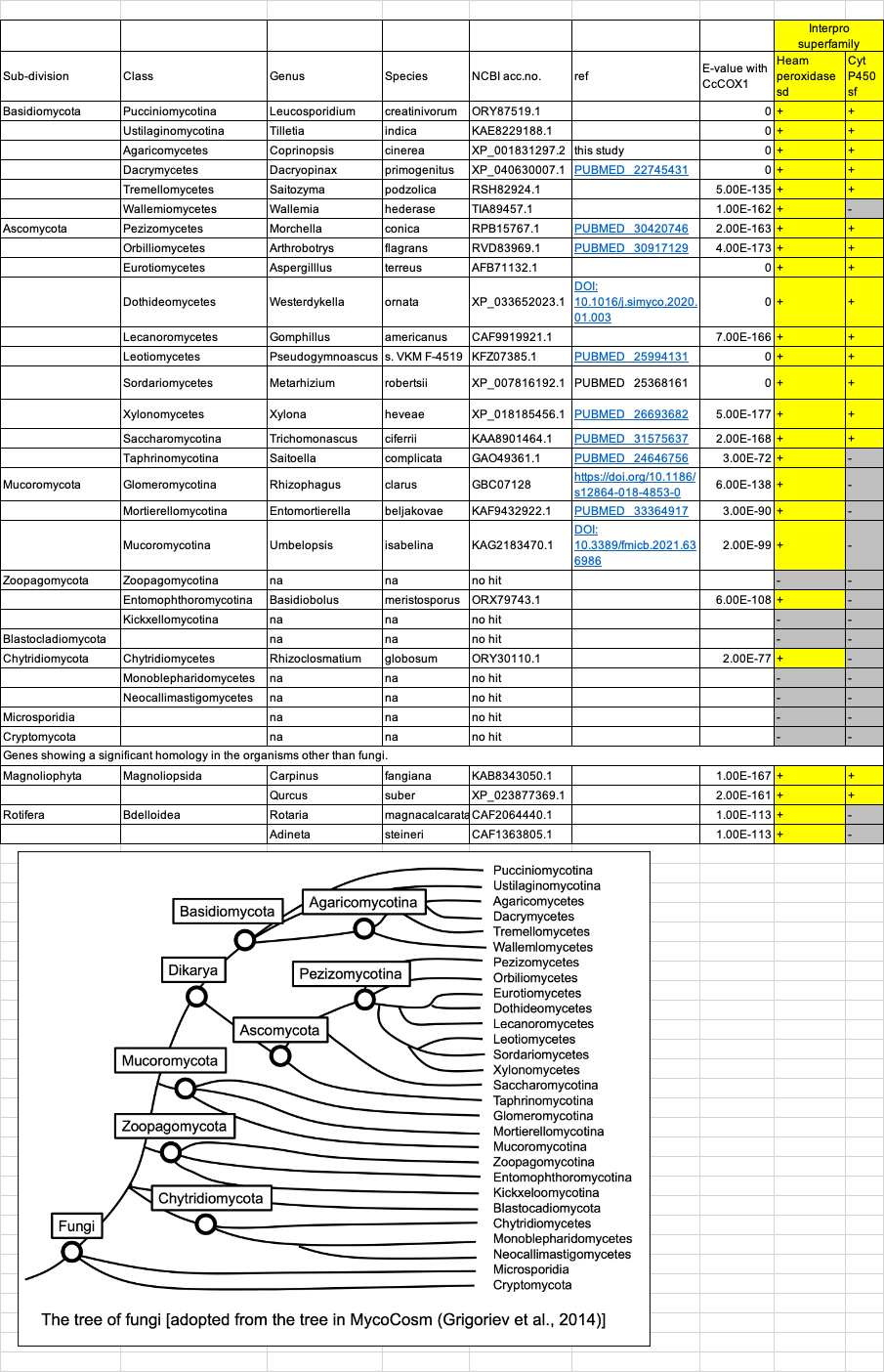


Table S4. Primers used in this study.


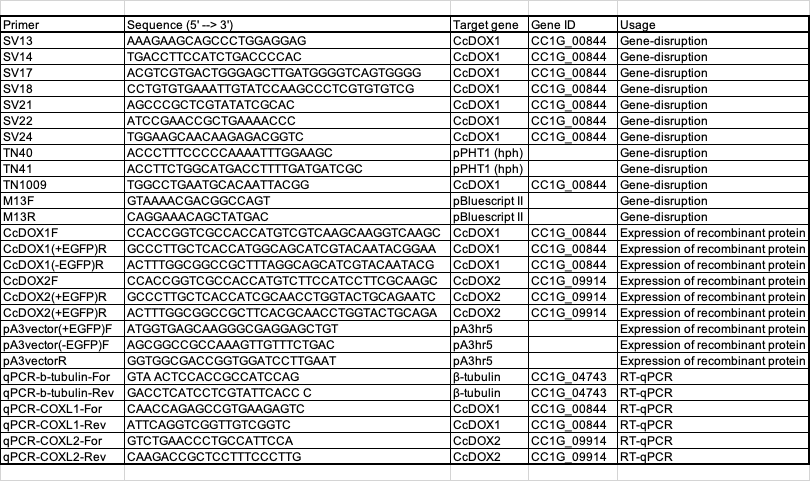

Supplement: Supplemental Figures S1–S15 and Tables S1–S4 [file mmc1.docx]
